# Case Report: Metastatic Parosteal Osteosarcoma in a Dog

**DOI:** 10.3389/fvets.2021.715908

**Published:** 2021-08-24

**Authors:** Sarah K. Samuels, Matthew R. Cook, Eric Green, Ryan Jennings, Roy R. Pool, Vincent A. Wavreille, William C. Kisseberth, Laura E. Selmic

**Affiliations:** ^1^Department of Veterinary Clinical Sciences, The Ohio State University, Columbus, OH, United States; ^2^Department of Veterinary Biosciences, The Ohio State University, Columbus, OH, United States; ^3^Department of Veterinary Pathobiology, Texas A&M University, College Station, TX, United States; ^4^Surgery Service, Small Animal Department, University of Zurich, Zurich, Switzerland

**Keywords:** canine (dog), bone tumor - osteosarcoma, metastases, parosteal or juxtacortical variant, osteosarcoma - pathology

## Abstract

This case report describes a rare form of malignant bone tumor in an 8-year-old Labrador retriever. This dog initially presented for evaluation of a right distal humeral mass. Radiographs of the right elbow and thorax were performed, revealing a smooth mineralized mass adjacent to the lateral aspect of the distal humerus and a 5mm pulmonary nodule. Computed tomography (CT) of the humerus and thorax showed a smooth mineralized lesion adjacent to the lateral humeral epicondyle, and a right cranial lung lobe nodule with a thin mineral rim. Surgical biopsies of both lesions were diagnostic for parosteal osteosarcoma (POSA). The dog was then treated with stereotactic body radiation therapy (SBRT) which controlled the dog's discomfort for 14 months until he became progressively painful and subsequently had his right forelimb amputated. This case report is the first to document the CT imaging characteristics of a metastatic appendicular POSA in a dog and the first dog described with POSA treated with SBRT. The dog lived for 623 days after histopathologic diagnosis and 849 days after initial presentation with pulmonary metastatic disease.

## Case Presentation

**Figure 1:**
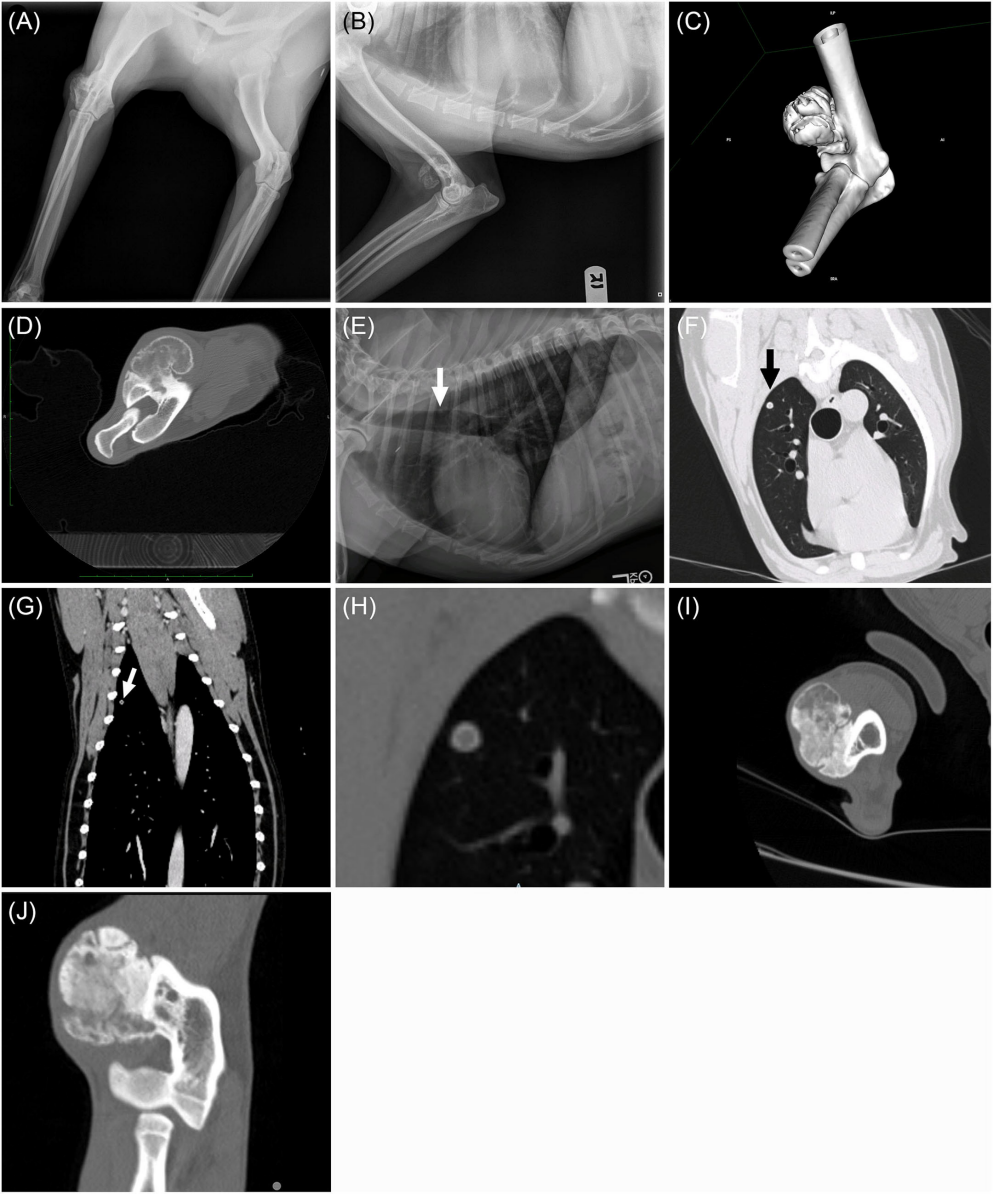
Craniocaudal **(A)** and mediolateral **(B)** radiographs of the right elbow. Note the smoothly marginated bony proliferation arising from the craniodistolateral humerus. Post-contrast transverse computed tomographic image of the right distal humerus. **(C)** Three-dimensional reformatted CT imaged displayed in a bone window and **(D)** lateromedial post-contrast transverse computed tomographic image of the right distal humerus. Note the lobular mineral proliferation arising from the craniodistolateral humeral metaphyseal cortex and lack of underlying humeral bone lysis. Left lateral thoracic radiograph **(E)**: 5mm rounded nodule within the dorsal third intercostal space (white arrow). Transverse lung window **(F)** and dorsal reformatted bone window **(G)** CT showing the pulmonary nodule within the right cranial lung lobe (black and white arrows). The thin, mineralized rim with a soft tissue center is evident on the enlarged transverse CT image displayed in a bone window **(H)**. Transverse **(I)** and dorsal **(J)** plane CT images in a bone window of the right elbow from repeat CT scan 217 days after completion of SBRT. Note that the soft tissue portions of the mass are more mineralized and the humeral cortex remains intact.

An 8-year-old male castrated Labrador retriever presented to The Ohio State University Veterinary Medical Center for evaluation of a suspected bone tumor of the right forelimb. The owners reported that the dog had a history of trauma to his right forelimb several years before presentation. The owners reported that the dog had been mildly lame on his right forelimb for about two months, and then a firm lump on the lateral aspect of his elbow was detected. The primary care veterinarian radiographed the elbow and a lobular, smoothly margined, mineral structure was noted on or adjacent the right distal humerus (Figures 1A,B).

At presentation, a 3 cm firm mass was palpated on the lateral aspect of the right elbow. Pain was elicited on full flexion of the right elbow, accompanied by resistance to joint flexion and discomfort on palpation and a mild lameness of the right thoracic limb. The right elbow was observed to be abducted at the walk and trot. The right prescapular and axillary lymph node were grossly normal on palpation. A complete blood count and serum biochemistry profile were performed and were unremarkable. Three-view thoracic radiographs were performed, and a 5 mm rounded soft tissue nodule was identified in the dorsal aspect of the right cranial lung lobe (Figure 1E).

Computed tomography (CT) was performed to further evaluate the elbow mass, characterize the pulmonary nodule better, and stage the abdomen for potential metastasis. CT scans of the elbow, thorax, and abdomen were performed under routine general anesthesia with Iohexol administration (2 mL/kg IV). The pre- and post-contrast CT images demonstrated a 3 cm, soft tissue and mineral mass arising from the craniodistolateral right humeral metaphyseal cortex (Figures 1C,D). The mass was in the region of the origin of the extensor carpi radialis and common digital extensor muscles. The humeral cortex was intact and dense periosteal proliferation arose from the cortex and served as the base for a lobular mass of mixed soft tissue and mineral. The margins of the lobules of the mass consisted of thin rims of mineral while the internal aspects of the mass were a mixture of soft tissue and faint, irregular mineral. The soft tissue portion of the mass enhanced following IV contrast administration. A round, smoothly marginated, well defined, 5 mm diameter nodule was present within the right cranial lung lobe (Figures 1F–H). The nodule had a thin mineral rim with a soft tissue center that did not contrast enhance. Several punctate mineral nodules were also present throughout the periphery of the lungs, consistent with pulmonary osteomas. No other pulmonary nodules were present. All abdominal structures and lymph nodes were unremarkable.

Based on the imaging diagnosis, the long-standing history of mild forelimb lameness, and the minimal clinical impact the elbow lesion had on the patient, the owners elected to monitor both the elbow and pulmonary lesion serially with imaging and empirically treated the mild discomfort in the elbow with carprofen (1.7mg/kg per os (PO) every 12 hours). The owners reported marked improvement in lameness with medical management and exercise restriction. Thoracic radiographs were repeated every three months. The pulmonary lesion increased 50% in size over the next six months while the right distal humeral lesion and lameness remained unchanged.

Based on the growth of the pulmonary nodule, surgical removal of the pulmonary nodule and incisional biopsy of the elbow mass were pursued 217 days after the initial presentation. A 5mm Michele trephine needle was used to collect an incisional biopsy of the bony lesion, and a thoracotomy and right cranial lung lobectomy were performed. After two days of hospitalization, the dog was discharged.

**Figure 2:**
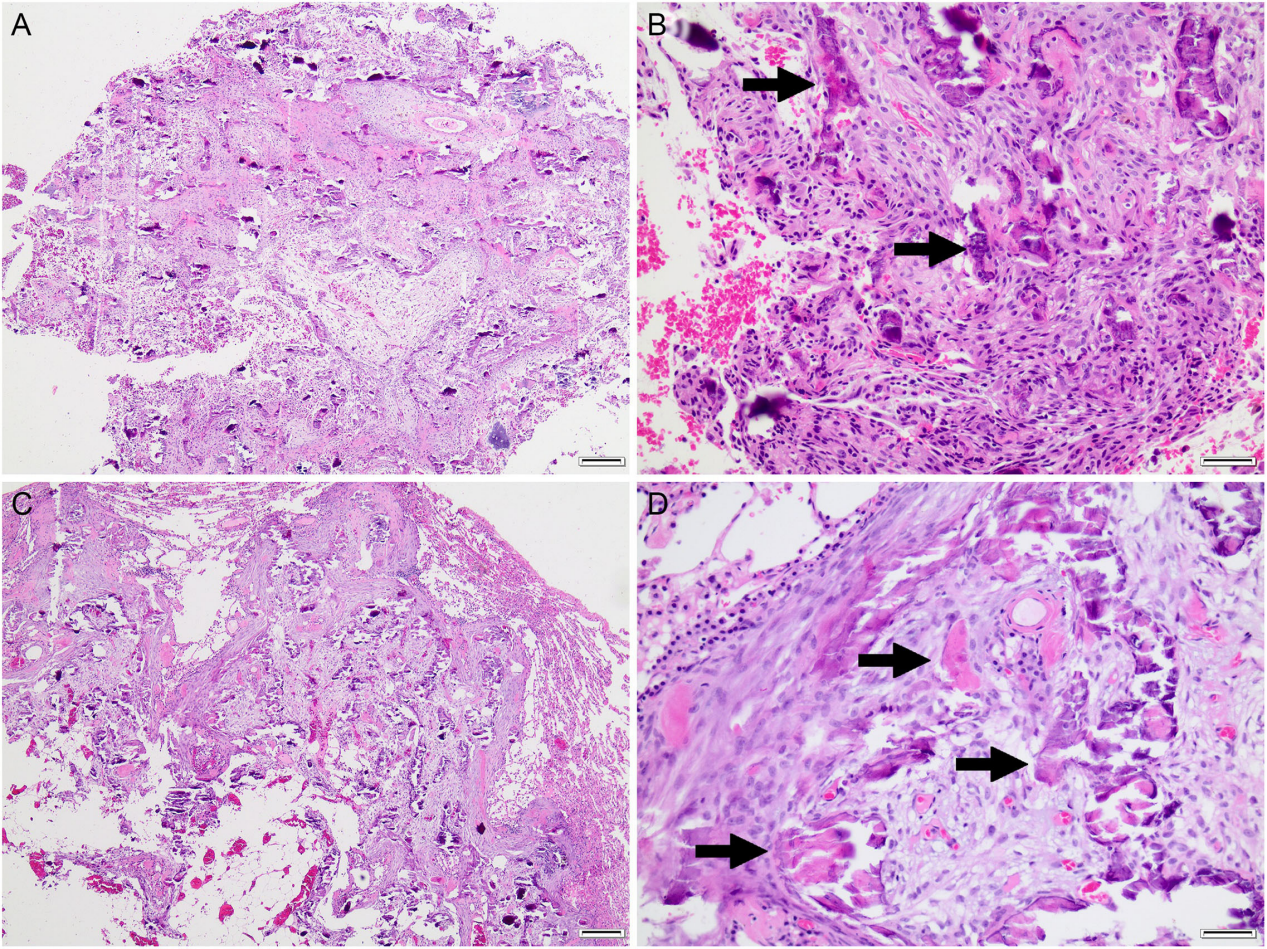
Histopathology of the right elbow mass **(A,B)** and pulmonary mass **(C,D)**. Both masses consisted of dense streaming bundles of mesenchymal cells interspersed with trabeculae of mineralized osteoid matrix (arrows) with scant woven bone and variable cartilaginous matrix (not shown). Hematoxylin and eosin. **(A,C)**, bar = 200 μm. **(B,C)**, bar = 50 μm.

Both the elbow mass and the pulmonary nodule were submitted for histopathology. Sections were examined by two anatomic pathologists. On histopathology (Figures 2A,B), both lung and elbow masses were discrete masses that contained foci of mineralized osteoid and woven bone formation associated with a population of spindloid mesenchymal cells with mild anisocytosis, mild anisokaryosis, and low mitotic activity. Within the lung nodule, the center of the mass contained well-differentiated adipocytes surrounded by an osteogenic population of mesenchymal cells, with osteoid deposition and woven bone. The elbow mass also had foci of cartilage formation (Figures 2A,B). The similarities between the osteogenic mesenchymal cell populations in the elbow and pulmonary masses were supportive of an osteogenic malignancy (Figures 2C,D). In the context of the radiographic findings, a diagnosis of POSA of the right elbow with pulmonary metastasis was favored.

Based on the histopathology, a diagnosis of metastatic POSA was established. Additional local and systemic therapies were recommended. As minimal bony lysis was present, and the dog continued to have good function of the forelimb, SBRT was completed 45 days after histopathologic diagnosis. A positioning CT scan was performed with the patient in left lateral recumbency, positioned within an immobilization mattress. The right forelimb was extended cranially and ventrally away from the neck and body. A 3D computer-based treatment plan was created based on the CT scan. The gross tumor volume (GTV) was defined as the mineralized mass. A 3 mm margin was placed around the GTV to define the planning target volume (PTV) but was limited to 2 mm along the lateral margin of the tumor to avoid including the dermis. No clinical target volume (CTV) was used. The source-axis distance plan consisted of four 6 megavoltage (MV) photon fields shaped to the PTV using the multileaf collimator (MLC). The dose was calculated using an anisotropic analytical algorithm (AAA). The treatment consisted of 3 doses of 10 Gy delivered in one week (Monday, Wednesday, and Friday) using a linear accelerator with a 160 leaf MLC. A megavoltage cone-beam CT was performed before each treatment to ensure accurate patient positioning. The locoregional lymph nodes were not treated with SBRT as they were normal in size. Following the treatment, no acute or late side effects, as per the Veterinary Radiation Therapy Oncology Group toxicity criteria, were noted (1).

After radiation therapy was completed, six doses of carboplatin were given (IV at 300 mg/m^2^ every 3 to 4 weeks). The dog had two asymptomatic adverse events during carboplatin treatment (one episode of grade 2 neutropenia and one episode of grade 2 thrombocytopenia) (2). Both resolved with chemotherapy delays Thoracic radiographs were repeated approximately four months after surgery and progressive disease (single 4 mm pulmonary soft tissue structure) was noted in the caudal thorax. Carboplatin chemotherapy was completed 187 days after histopathologic diagnosis.

A repeat CT scan of the elbow was performed 218 days after radiation therapy (261 days after histopathologic diagnosis) and showed that his distal humeral mass was static in size (4.7 cm in longest dimension) compared to his pre-radiation therapy CT scan (5.0 cm in longest dimension). The soft tissue portions of the mass were more mineralized and there were still no changes to the humeral cortex (Figures 1I,J). Additionally, chest radiographs were repeated. While the previously observed soft tissue thoracic nodule in the caudal thorax was not observed, two small pulmonary nodules were observed and were concerning for additional pulmonary metastatic disease. After completion of carboplatin chemotherapy, metronomic chemotherapy was recommended for management of the dog's metastatic disease. Seventy-four days after the completion of carboplatin chemotherapy (261 days after histopathologic diagnosis), the owners elected to start the dog on metronomic chlorambucil (3 mg/m^2^ PO every 24 hours). Chlorambucil was well tolerated and no adverse events were observed with its administration.

Five hundred and four days after histopathologic diagnosis, the dog developed a moderate mechanical lameness. Repeat radiographs of the right elbow were performed and the mass appeared minimally larger than previously measured (5.1 × 4.4cm). All peripheral lymph nodes palpated within normal limits on physical exam. Repeat chest radiographs were also performed demonstrating minimally progressive pulmonary metastatic disease. Chlorambucil was discontinued prior to amputation after the dog had received metronomic chlorambucil for 243 days. Right forequarter amputation and extirpation of the right axillary lymph node was then performed without complication. Histopathology of the right forelimb was consistent with a moderately differentiated chondroblastic parosteal osteosarcoma. Mild anisocytosis and anisokaryosis was observed. 5 mitotic figures were seen in ten 400 × fields. No significant microscopic findings were identified in the right axillary lymph node. Local invasion of the neoplasm was observed into the adjacent bone.

Two months after amputation of his primary tumor, repeat chest radiographs were performed and a 10 × 8 × 7.8 cm accessory lung lobe mass was identified with progressive pulmonary metastatic disease. The 10cm pulmonary mass was sampled with ultrasound guidance and was cytologically diagnostic for a sarcoma. Alkaline phosphatase immunocytochemistry was performed on the cytology sample and positive staining of the neoplastic cells was reported. The dog was started on toceranib phosphate (PO at 2.54 mg/kg every Monday, Wednesday, and Friday).

Fifty-six days after starting toceranib (569 days after histopathologic diagnsosis), progressive coughing and lethargy was reported by the owner. Repeat chest radiographs were performed and progressive pulmonary metastatic and pleural effusion was observed and toceranib was discontinued. The dog was euthanized 632 days after histopathologic diagnosis after beginning to have trouble breathing. SBRT managed to control the dog's discomfort associated with the tumor for 461 days until progressive lameness was noted and amputation was perused. Additionally, the dog lived 588 days after the completion of SBRT.

## Discussion

This case report describes the imaging characteristics and outcome of a dog with metastatic appendicular POSA. This tumor was found in the right distal humerus, and, at the time of initial diagnosis, a pulmonary nodule was identified in the right cranial lung lobe. To the authors' knowledge, this is the first reported case of POSA in the appendicular skeleton of a dog with stage III disease at the time of presentation and a good long-term outcome with a survival of over 20 months with multimodal therapy and continued good function of his right forelimb. Additionally, this was achieved without amputation or primary surgical excision of his POSA.

One previous case report from 1971 describes a German Shepard dog who had POSA of the carpus that was diagnosed based on a correlation between imaging with radiographs and incisional biopsy (3). The dog was treated with Cobalt-60 teletherapy over a 1.5-year period (3). Approximately 1.5 years after treatment, thoracic radiographs were performed, pulmonary metastatic disease was detected, and the dog was euthanized (3). While this case highlights the indolent nature of this poorly reported disease, pulmonary metastatic disease was not detected for approximately 18 months from the time of diagnosis. In the case presented here, a similar survival time was achieved with stage III disease being present at the time of diagnosis that was treated with metastasectomy, SBRT, and carboplatin chemotherapy. This suggests that patients with POSA with stage III disease may have longer outcomes in comparison to dogs with more conventional osteosarcoma.

Parosteal osteosarcoma forms in the fibrous layer of the periosteal connective tissue of bone and is composed of a hypocellular stroma of spindle cells, trabeculae of bone, and a cartilaginous component (4–6). This is a slow-growing tumor arising from the periosteum and, in humans, is typically less likely to metastasize and, therefore, usually has an overall better prognosis compared to more common high-grade osteosarcomas (5–7). If left untreated, this tumor can dedifferentiate into a more aggressive malignancy, extending into the cortex and invading the medullary cavity (8–11). This dedifferentiation is well described in humans but has yet to be demonstrated in dogs (8–11).

Parosteal osteosarcomas are reported to appear on radiographs as lobular, opaque masses with a smooth border arising from the surface of the bone in dogs (5). In humans, the radiographic features have been more extensively described, where POSA are typically lobular masses arising from the underlying cortex with an irregular pattern of mineralization (12, 13). Some have been reported to have a long base of attachment extending along the periosteum (7, 12). In contrast to animals, the margins of these tumors were reported to be irregular due to linear extensions into the soft tissues (14). A region of linear lucency isolating the mass from the neighboring bone is a reliable diagnostic feature in humans, though it is not always present (13, 15, 16). This linear lucency was not present in the radiographic images of the dog in this report.

In humans, imaging can be useful in distinguishing low-grade POSA from higher grade osteogenic malignancies or de-differentiated POSA (17). On CT imaging, POSA often have characteristic radiolucent areas within these calcified tumors in addition to satellite nodules. Low-grade POSA lesions are often smaller than higher-grade or de-differentiated lesions, often do not have pulmonary metastatic disease, and often lack of a *sunburst* periosteal reaction on imaging (17). Additionally, more aggressive or dedifferentiated POSA will often have an ill-defined soft tissue mass associated within or adjacent to the primary ossified tumor. Low-grade tumors have also been described as having an osteoid matrix of high attenuation that was greater than underlying intramedullary bone (12).

Computed tomography and magnetic resonance imaging have also been used in diagnosing POSA. It is helpful in accurately determining the local extent of the mass for potential surgical planning. These imaging modalities can assist in planning therapy since they can help give information about the potential biological grade of the tumor (16). There are minimal descriptions of the CT appearance of POSA in dogs (6). The dog described here is the first appendicular POSA described and imaged with CT. While metastatic disease was present at the time of initial presenation, this dog lived 623 days after histologic diagnosis, suggesting that metastatic disease alone may not be a strong predictor of outcome for dogs with POSA. Though the dog had slowly progressive metastatic disease despite chemotherapy, the dog lived significantly longer than dogs with stage III central osteosarcoma where the median survival time for dogs with stage III metastatic disease have a median survival time of 49 days (18). While some dogs may be cured with surgical excision of their primary bony tumor, metastatic disease may be a relatively poor prognostic indicator for dogs with POSA. Additionally, no *sunburst* periosteal reaction was observed on imaging, suggesting the lesion may have been more consistent with a low-grade POSA (13, 15).

This case described here is the first case of a POSA managed multimodal therapy with SBRT, adjuvant chemotherapy, and limb amputation. In a recent publication exploring the outcomes of 123 dogs with central appendicular osteosarcoma treated with SBRT, the reported time to first event was 143 days and the overall median survival time was 233 days (19). Additionally, 15 dogs with appendicular osteosarcoma treated with SBRT that had metastatic disease at the time of treatment were reported to have a median survival time of 200 days, though histopathology or cytologic diagnosis of these dogs' metastatic disease was not documented in this publication (19). Outcomes of dogs with POSA treated with radiation therapy may be significantly improved compared to dogs with central OSA based on its more indolent biologic behavior, though there is currently a paucity of publications evaluating clinical outcomes of dogs with POSA treated with radiation therapy. ‘This case suggests that SBRT with adjuvant chemotherapy may be a reasonable treatment for primary and metastatic POSA in select cases.'

Currently, very little is published on the histopathology of parosteal and surface osteosarcoma in dogs. To date, only two studies have evaluated the histopathology of POSA in dogs; one case series evaluating zygomatic POSA in dogs and one cat and one small retrospective case series of dogs with POSA and periosteal osteosarcoma (19, 20). In the case series evaluating tumor grade and patient outcomes of dogs with POSA and surface OSA, primary tumor grade did not correlate with survival (20). One limitation of this case report was that Ki-67 was unable to be performed. Additional research is needed to expand on the histology of POSA and surface OSA in dogs.

In addition to the primary bony lesion, the metastatic nodule in the dorsal aspect of the right cranial lung lobe was also imaged with radiographs and CT. This nodule had a thin mineral rim with a soft tissue center without central contrast enhancement. Additionally, adipocytes surrounded by mesenchymal cells were observed within the pulmonary metastatic lesion on histopathology. While unusual, about 15% of POSA in humans are reported to have focal fatty components between trabecular bone (11).

## Conclusion

This case report describes the first reported appendicular POSA with concurrent and progressive pulmonary metastasis in a dog treated with multimodal therapy and a good long-term outcome with the dog living 623 days from the date of histologic diagnosis. This tumor type should be considered a differential diagnosis for a bony mass without concurrent lysis, particularly if it fits with the CT imaging and histopathologic characteristics described above. The clinical trajectory in this dog mirrors what we know about POSA in humans, where patients have more prolonged survival compared to conventional osteosarcoma. This is the first reported case of a canine POSA treated with SBRT. SBRT should be considered as a potential treatment option for dogs with POSA, as it helped manage his discomfort when ambulating for 15 months. This is also the first case of stage III POSA described in a dog with a long-term survival with multimodal treatment.

## Data Availability Statement

The original contributions presented in the study are included in the article/supplementary material, further inquiries can be directed to the corresponding author/s.

## Author Contributions

SS wrote the manuscript. LS assisted in supervision of the clinical management of the case and contributed to conception of the case report. MC contributed to the conception of the case report and assisted in primary case management. VW and WK supervised the clinical management of the case. RP and RJ assisted in pathology reviewing. EG assisted with the imaging and assisted in supervision of the clinical management of the case. All authors critically reviewed and approved the final version of the manuscript.

## Conflict of Interest

The authors declare that the research was conducted in the absence of any commercial or financial relationships that could be construed as a potential conflict of interest.

## Publisher's Note

All claims expressed in this article are solely those of the authors and do not necessarily represent those of their affiliated organizations, or those of the publisher, the editors and the reviewers. Any product that may be evaluated in this article, or claim that may be made by its manufacturer, is not guaranteed or endorsed by the publisher.
